# Calculi

**Published:** 1829-06

**Authors:** 


### CALCULI.

*Calculi removed from the Bladder by the Urethral Forceps; Death.*
(Guy's Hospital.)

J. Money, aged sixty, admitted December 24th, 1828. He states that eight years ago he began to void with his urine a deposit of fine sandy matter: the particles forming this deposit gradually became larger, until at length the constant discharge of sand was succeeded by the occasional escape of small calculi, which varied in size from that of a barleycorn to a large pea. During the last twelve months no calculi have been discharged; at the same time the sufferings of the patient, from distinct symptoms of stone in the bladder, have become very severe. The only common symptom absent in this case is the abrupt stop to the stream of urine during its discharge.

On sounding the patient soon after admission, Mr. Key ascertained that the symptoms depended on numerous calculi, rather small in size: he thought the bladder might contain twelve or fifteen of them, and that their size probably admitted of removal by the urethra, which was the more desirable as the patient's ill state of health entirely forbad the operation of lithotomy. His appearance was that of a previously hale man, now suffering severely from disease: his system was feverish and irritable from constant pain, his breathing difficult, cough frequent, pulse irregular and intermitting; every slight cold excites disorder of the lungs, and occasionally he complains of pain in the region of the kidneys.

December 24.—℞ Liquoris Potassæ gt. xx.; Tinct. Hyoscyami gtt. xl.; Aquæ Menthæ ℥iss. Omni nocte sumend.

January 8.—An accession of fever, with aggravation of chest affection.—Pil. Colocynth. Calomelanos gr. x. statim.

Jan. 26th.—No material or permanent improvement.—℞ Potas. Carbonatis ℈ij.; Potas. Nitratis gr. x. bis in die sumend.—℞ Ext. Conii gr. v. omni nocte sumend.

The above and similar treatment was pursued until March 9th, with more or less relief to the urgency of his symptoms.

March 9th.—He suffers much less pain, and his system generally is in a more tranquil condition; but is not in a state to submit to the usual operation, with any hope of recovery. Mr. Key therefore determined to try what could be done in removing the calculi per urethram. The facility with which this, in the first attempt, was accomplished, and the very little pain it gave the patient, were very pleasing. The instrument, scarcely thicker than a good-sized sound, was introduced almost as readily as the sound generally is, and was as quickly withdrawn, with a stone in its grasp. This was done three times, with no other difficulty than that, in withdrawing one of the stones, it hung a little in the narrow part of the canal, close to the external orifice. Mr. Key did not introduce the forceps a fourth time, leaving the remaining calculi to future attempts, that the amount of irritation might be divided. The stones were polished and angular, about the size of common plum stones, being larger than any the patient had previously voided. His gratitude on finding three of his tormentors removed with so little pain was very clearly expressed. In the afternoon a fourth calculus, nearly equal in size to the others, spontaneously came away with his urine. No constitutional irritation followed this operation, and up to March 28 the patient's health continued very nearly as before.

March 28th.—The forceps were again used. On this occasion the calculi were less readily grasped, and with more difficulty removed. Of one Mr. Key was compelled to relinquish his hold just anterior to the scrotum, but by external pressure he succeeded in forcing it along the urethra, and finally in expelling it. A second was removed also with difficulty, especially at the anterior contraction of the canal, where it long resisted both pressure from behind and the application of common forceps through the orifice. The removal of these two calculi having been attended by great pain and some bleeding from the urethra, further attempts were refrained from, and the patient put to bed.

30th.—Since the operation, the patient has suffered severe and almost constant pain in the urethra and neck of the bladder, it being especially acute whenever he voids urine. There is also considerable disturbance of the system: the pulse is quick and irregular, cough troublesome, breathing harassed, and general febrile symptoms.

April 6th.—The case becomes serious: the local pain increases, and is described by the patient as exactly resembling the sensation of a calculus lodging in the commencement of the urethra; but the introduction of a sound does not discover one. Urine very turbid, containing much mucus. The disturbance of the system is fully proportioned to the local suffering.

11th.—Alarmingly worse. Pain in the neck of the bladder and perineum is extreme; no tumor appears externally. The patient is fast wearing out; his countenance becomes sunken; pulse very quick and weak; mouth parched, tongue dry and brown; cough frequent and very distressing. The urine is exceedingly unhealthy, as if mingled with dark-coloured fetid pus.

12th.—This morning a most distressing attack of bilious vomiting supervened, with increasing weakness and exhaustion. Sir A. Cooper saw him today, and prescribed, but it was evidently in vain. The poor fellow expired the following day.

*Inspectio cadaveris.*—The bladder contained sixteen calculi, varying from the size of a horsebean to that of a large walnut. Its coats were thickened and contracted; the mucous membrane of a dark gray or ashy colour; and the fluid in the cavity was thick, dark-coloured, and offensive. The ureters were dilated to three or four times their natural caliber; their lining membrane, as well as that of the pelvis of the kidney, was softened, and had the same ashy colour with the bladder: they also contained the same semi-purulent fluid. The membranous portion of the urethra, immediately behind the triangular ligament, was deeply discoloured, as if from slough or gangrene; and a very small opening was found leading from it to a cavity, apparently the collapsed cyst of an abscess, situated within the pelvis, between the bladder and rectum, rather to the right side: this contained a similar fluid to that found in the bladder, and its parietes were ragged and of the same dark ashy colour. The other viscera presented no recent or active disease: the lungs were nearly healthy, the heart above the natural size, the aorta dilated, and at some points having patches of ossification.*

